# Diagnosing Medication Non-Adherence in a Patient with Myocardial Infarction

**DOI:** 10.3389/fpsyg.2012.00267

**Published:** 2012-08-03

**Authors:** Siqin Ye, David J. Krupka, Karina W. Davidson

**Affiliations:** ^1^Center for Behavioral Cardiovascular Health, Department of Medicine, Columbia University Medical CenterNew York, NY, USA

**Keywords:** medication non-adherence, assessment, cardiovascular diseases, chronic disease management

## Abstract

**Background:** Medication non-adherence continues to be a major challenge facing the healthcare system. A case is presented of a 48-year-old man with myocardial infarction who was found to be non-adherent to multiple medications. Conceptual models are reviewed along with current approaches for assessment and treatment of medication non-adherence. **Design:** Case report and literature review. **Discussion:** A theoretical model for medication non-adherence built on the Theory of Planned Behavior is presented. Empirical evidence is reviewed for determinants of non-adherent behavior such as health beliefs and self-efficacy. Current methods to assess medication non-adherence, including self-report, pill count, biological drug levels, pharmacy refill, and electronic bottles are summarized along with their limitations. Finally, an individualized approach for assessment is described using the case presented and the conceptual framework outlined above. Follow-up for the patient and potential interventions to improve medication adherence are discussed. **Conclusion:** Despite the challenges, a conceptual framework for medication non-adherence can guide assessment and treatment. Further research for innovative and effective methods to detect and treat medication non-adherence is urgently needed to aid clinicians in treating this pervasive behavioral problem.

## Introduction

Mr. P is a 48-year-old African American patient with a history of end-stage renal disease and ischemic cardiomyopathy status post coronary artery bypass grafting who presented to our hospital with a myocardial infarction (MI). At admission, he reported that he had discontinued the aspirin and simvastatin he was supposed to be taking at home, though he later stated that he took all his medications regularly. He had also intermittently missed several dialysis sessions, and had missed a scheduled nuclear cardiology test the week prior to admission. During his hospitalization, he initially agreed to a cardiac catheterization procedure, then changed his mind and refused. He was also observed eating a snack high in sodium during a dialysis session despite education on sodium restriction. When questioned, he denied that this occurred. After 2 days in hospital, he was discharged on nine medications.

At the time of admission, Mr. P consented to participate in a research project assessing medication adherence. As part of the research protocol, he answered a brief series of questionnaires with regards to his socioeconomic background. He was a high school graduate, and worked as a certified construction worker until he was diagnosed with renal insufficiency, after which he became a security guard. He had been unemployed since 2000, and reported an annual salary of $5,000. He was eligible for and received Medicaid. Otherwise, he lived alone, but reported having daily contact with his mother, who also had end-stage renal disease. He had an 18-years old son, with whom he had no contact for many years.

## Background

### Why should health psychologists care?

The pattern of behavior demonstrated by Mr. P raises concerns of medication non-adherence. Medication non-adherence, defined here as the failure to take medications as prescribed, is arguably one of the largest behavioral challenges facing the health care system today (Osterberg and Blaschke, 2005). Despite several decades of research that has highlighted the prevalence and negative impact of medication non-adherence, there remain significant barriers to adequately detect and treat this pervasive behavioral problem (Osterberg and Blaschke, 2005; Rasmussen et al., 2007). As will be discussed below, the complex reasons for medication non-adherence and the optimal methods to assess it are yet to be fully elucidated, a process that will require considerable contribution from the theory, research, and practice of the field of health psychology.

### Determinants of non-adherence

**Figure 1:**
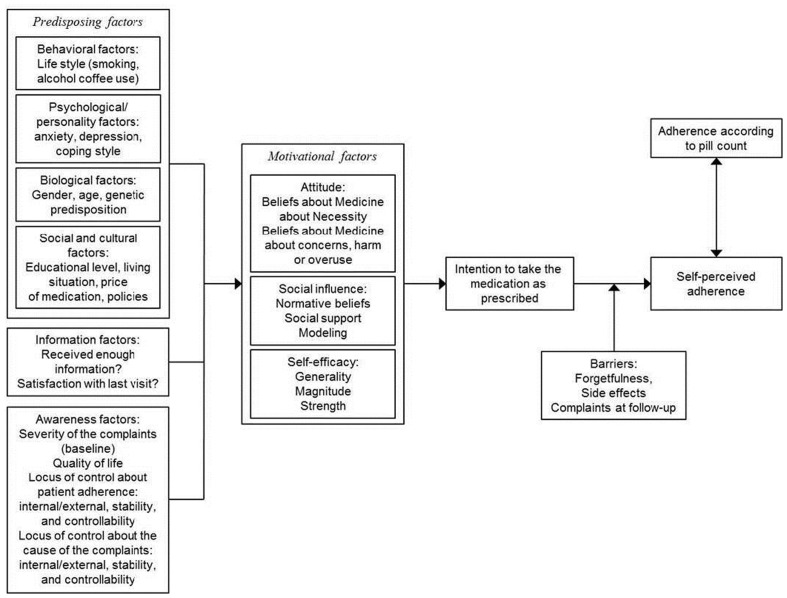
**Theoretical model: determinants of patient adherence**. Adapted from “Which patient-related factors determine self-perceived patient adherence to prescribed dyspepsia medication?” by Fransen et al. (2009). Copyright 2009 by G. A. J. Fransen. Reprinted with permission.

A number of theoretical models for health behavior have been specifically adapted for the phenomenon of medication non-adherence. These include the Health Beliefs Model, that posits that adherent behavior is predicated upon patients recognizing the potential adverse consequences of non-adherence; the Theory of Reasoned Action, that adds to the Health Beliefs Model by incorporating subjective norms such as those of family members and providers that promote adherence; and the Theory of Planned Behavior, that further incorporates self-efficacy and perceived barriers into the model to account for factors that may or may not lie within the patient’s locus of control (Becker, 1974; Ajzen, 1991; Ryan, 1998). More robust models also recognize additional and complementary causes of medication non-adherence at various levels of analysis, including the patient-provider relationship, systemic barriers such as lack of access and financial resources, and the broader social-economic and cultural context that may impact the perception of medication efficacy and side-effects. An example of such a comprehensive schema that combines the theoretical models mentioned above is provided by Fransen and others (Figure 1). The model summarizes the multiple potential determinants of medication non-adherence and how they inform health beliefs and perceptions of self-efficacy and social norms, to culminate in the intentional behavior of medication taking (Fransen et al., 2009).

The empirical literature regarding determinants of non-adherence has tended to focus on various individual components of the comprehensive model. For instance, studies using pharmacy refill data have reliably demonstrated the impact of higher medication copayment on non-adherence (Schneeweiss et al., 2007), while other studies have demonstrated that demographic and socioeconomic factors including age (both older and younger), female gender, and lower income status are associated with non-adherence (Brenner et al., 2002; Rasmussen et al., 2007). Medication non-adherence has also been noted to be particularly problematic in individuals with chronic kidney disease, such as Mr. P (Magacho et al., 2011). Consistent with the theoretical models described above, health beliefs about the relative benefit and potential adverse outcomes of medications have been linked to non-adherence across different diseases (Horne et al., 1999, 2010), as has self-efficacy (Littlefield et al., 1992). Also of interest to health psychologists in particular may be the relationship between non-adherence and psychological traits. For instance, both increased level of hostility and decreased level of conscientiousness are associated with medication non-adherence (Zugelj et al., 2010; Farrell et al., 2011). Finally, there is an emerging body of literature highlighting the elevated risk of medication non-adherence in cardiovascular patients who have depressive symptoms, and it is hypothesized that non-adherence may be a potential mediator of the known association between depression and adverse cardiovascular outcomes (Brenner et al., 2002; Rieckmann et al., 2006; Compare et al., 2011).

### Assessment of medication adherence

Despite these insights, thus far there remains little consensus on standardized and practical approaches for assessing medication non-adherence. In part this is due to the lack of reliable, validated measurement methods that are useful in both research and clinical settings. Currently, the most commonly used approaches include self-report, pill counts, biological drug levels, pharmacy refill data, and electronic pill bottles, each of which has shortcomings (Osterberg and Blaschke, 2005). Although self-reports of medication non-adherence can be easily to obtained, both informally or through use of standardized instruments such as the Morisky scale (Morisky et al., 2008), the accuracy of this approach is often questioned due to significant underreporting (Ho et al., 2009; Mann et al., 2010). Pill counting, a more objective measure, tends to be more time consuming and fails to capture nuances of medication taking behavior (Cramer et al., 1989; Osterberg and Blaschke, 2005). Biological surrogates, such as blood and urine drug levels or intermediate indices like serum cholesterol or glycosylated hemoglobin that reflect the target of medications, are more costly and have limited applicability to the broad range of medications that are commonly used (Osterberg and Blaschke, 2005; Parris et al., 2005). In recent years, the availability of large databases of insurance claims and pharmacy refills has given rise to new methods for detecting non-adherence. These include measures such as the medication possession ratio, calculated by dividing the total number of doses dispensed by the total number of doses that should have been taken during the days monitored (Taira et al., 2006; Chan et al., 2010). To date, however, there exist considerable infrastructural barriers in applying this methodology to individual patients, given that closed pharmacies and centralized electronic medical records are only available in a limited number of integrated healthcare systems (Ho et al., 2009). A more promising approach is the use of devices such as electronic pill bottles, which are able to capture each instance of medication taking behavior and allow the analysis of processes by which behavioral tendencies to adhere or not are established (Osterberg and Blaschke, 2005; Rieckmann et al., 2006). Although the effectiveness of this method is now well-proven in research settings, issues of cost, ease of use, and patient acceptance remain barriers for more wide-spread clinical application.

Because of these limitations, there remains considerable need for further research on optimal methods to assess medication non-adherence. As prior research has mostly focused on assessment of non-adherence to individual medications for specific conditions, there should also be more emphasis on elucidating potential behavioral patterns of non-adherence that persist across medication classes. Further research is also needed to establish a consensus for standardized measurement tools that can be generalized to clinical settings, and that will be useful for both patients and providers (Ho et al., 2009). Nonetheless, given the high prevalence of non-adherence and the associated burden of adverse outcomes, medication non-adherence should be assessed routinely while awaiting answers from ongoing research. An example of approaches to and challenges of assessing medication adherence will be discussed using the example of Mr. P.

## Discussion

### Assessment of medication non-adherence in research setting

Because Mr. P was enrolled in a research protocol, he underwent a multifaceted assessment of medication adherence behaviors and potential barriers, using instruments informed by the comprehensive model described above. He was first screened for potential cognitive and psychological conditions that might negatively impact adherence, using questionnaires that included the Mini-mental status exam (Folstein et al., 1975), an interview screen for psychosis and other major mental disorders, the Alcohol Use Disorders Identification Test (AUDIT; Saunders and Aasland, 1987), and a substance abuse survey. Mr. P screened negative for all of these. He then completed another set of questionnaires about his medical history, employment status, education level, and social support. For depressive symptoms, the Beck Depression Inventory (BDI) version I (Beck et al., 1961) was used because it was known to predict both mortality and medication non-adherence in this patient population (Rieckmann et al., 2006; Lichtman et al., 2008). At the time of his hospitalization as well as 1 month later, his depressive symptoms were in the moderate range on the BDI (a score of 14). To specifically assess medication adherence, Mr. P completed a phone interview at 1 week after discharge during which he was asked about his ability to pay for his medications and any potential barriers for filling his aspirin, metoprolol, clopidogrel, and simvastatin prescriptions. He also completed the Morisky eight-item self-report medication adherence scale for each of the four medications (Morisky et al., 2008). At the 1-month visit, he was assessed with the modified Coronary Artery Risk Development in Young Adults (CARDIA) medication adherence scale, which quantifies the number of days within the past week on which the monitored medications were taken (Cutter et al., 1991). Finally, an objective assessment of adherence was obtained using an electronic pill bottle that had four compartments, one for each of the monitored medications.

The results of assessment for Mr. P are displayed in Table A1 in Appendix. At the time of discharge, Mr. P reported that he did not foresee difficulties in obtaining or filling his prescriptions, and that he was able to afford his medications. At 1 week, he reported that he had filled all four of his prescriptions. On the Morisky eight-item self-report medication adherence scale, Mr. P received a score of 2 out of a possible 8 for all four medications, which is classified as “poor adherence.” At the 1-month follow-up visit, he reported on the modified CARDIA medication adherence scale that he had taken his aspirin, metoprolol, and simvastatin every day for the past week as prescribed, which was consistent with a score of 100% for self-reported adherence. However, electronic pill bottle monitoring demonstrated that his self-reported adherence rate at 1 month was a vast overestimate, as he only opened the aspirin compartment of the pillbox on 6 out of 31 days (19.4%), and the metoprolol and simvastatin compartments on 2 out of 31 days (6.5%) and 3 out of 31 days (9.7%), respectively. He was also found to have never opened the clopidogrel compartment, and when questioned, he first insisted that he had filled the clopidogrel prescription, and then eventually admitted that he had in fact not done so.

Mr. P had also completed the Beliefs about Medications – General Beliefs – Questionnaire (Horne et al., 1999). He had a high overall score, indicating distrust. Furthermore, on specific items he indicated that he strongly agreed that “doctors place too much trust in medicines… if they had more time with patients, they would prescribe fewer medications.” Also, he indicated that “most medicines are addictive,” and questioned whether “medicines do more harm than good.” Taken as a whole, it was apparent that although Mr. P reported only intermittent non-adherence to his medications, his medication taking behavior as objectively measured through electronic pill bottle monitoring was poor, and he endorsed beliefs about medications being overused and being potentially harmful that are consistent with his actual behavior.

### Individualized approach to medication assessment

Although the research protocol described above necessarily differs from how one might assess medication non-adherence in clinical practice, a number of insights can be drawn and applied. Current expert consensus recommends that despite the limitations of self-report, asking about medication non-adherence in a direct and non-judgmental fashion should be a routine component of clinical visits (Osterberg and Blaschke, 2005; Ho et al., 2009). In the case of Mr. P, this likely would have raised high suspicion for medication non-adherence, given the different answers he gave to different providers. Furthermore, confirmation for medication non-adherence can be achieved by additional methods such as asking patients to bring medication bottles from home, or by calling outpatient pharmacy to confirm if important medications have been filled. When used judiciously for patients in whom there is heightened concern for non-adherence, these approaches would not be overly burdensome for most providers. At the same time, health literacy and health beliefs about specific medications and medical conditions can be explored, and self-efficacy and other potential barriers such as costs of medications and access to the healthcare system should be assessed. This can be done in an informal manner with open-ended questions. By helping to uncover reasons for medication non-adherence that could be promptly addressed, the additional time required would also likely represent time well-spent to improve patient outcomes.

### Outcome of assessment

Because the research protocol was focused on assessment of medication non-adherence, clinical outcomes were not routinely collected. Nonetheless, Mr. P gave permission to contact his outpatient cardiologist, Dr. K, after the completion of the study protocol. More than a year has since passed, and although Mr. P did not have any further cardiac events, he continued to miss many appointments. In part because of his non-adherence to medications, his hypertension remained poorly controlled, and he still reported intermittent chest pain, with an adverse impact on his quality of life. There was also concern that his coronary artery disease may have further progressed. A recent repeat stress test showed new areas of cardiac ischemia, but despite these high risk features, he again missed his follow-up cardiology appointment. Dr. K stated that he has also suspected medication non-adherence, but has been unable to further engage with Mr. P to make healthy behavioral changes or to take his medications more regularly. “It is very frustrating,” Dr. K said, “but given the limited time and resources, it is very difficult to find out why he does not take his medications and convince him otherwise.”

### Potential interventions for behavioral change: From theory to practice

Dr. K’s comments underscore the common perception that changing non-adherent behavior is a frustratingly difficult endeavor. Health psychologists, with their expertise and unique insights into the behavioral determinants of health outcomes, are well-positioned to facilitate assessment and treatment of complex medication non-adherence issues as exemplified by Mr. P. Although this review is primarily focused on assessment of non-adherence, treatment options will be briefly discussed. As a rule, the comprehensive conceptual model presented above can be used as the framework to guide treatment strategies.

Given the core importance of health beliefs and perceived self-efficacy as key determinants of medication adherence, it is not surprising that the majority of the interventions that have been studied have focused on these aspects. For instance, one study of patients with MI using educational mailings that emphasized the benefit of beta-blockers and suggested ways to deal with side-effects showed improved beta-blocker adherence (Smith et al., 2008). Other interventions to provide similar education and reinforce self-efficacy have included phone calls (Rudd et al., 2004; Marquez Contreras et al., 2005) or in-person sessions with pharmacists (Lee et al., 2006; Murray et al., 2007). In general, more intensive interventions have tended to be more successful. Considerations of other barriers to adherence have led to additional approaches, ranging from an ongoing trial to promote adherence through complete elimination of copays (Choudhry et al., 2008), to technological solutions such as text message reminders on mobile phones (Lester et al., 2010). For most of these interventions, however, their applicability and cost-effectiveness in the general population remain to be determined.

Because no standardized adherence interventions exist, an understanding of why a particular patient is non-adherent becomes essential for guiding the choice of treatment. In Mr. P’s case, for example, it is apparent that a major cause of non-adherence was his distrust of the medical system, which led him to discount the efficacy and exaggerate the harms associated with his medications. It is essential therefore for his providers to engage with him about his health beliefs so as to improve therapeutic alignment. Where the resources exist, focused interventions by health psychologists using counseling techniques such as motivational interviewing or problem solving therapy can contribute greatly to such efforts (Resnicow et al., 2002). In addition, Mr. P’s interview revealed that he lived alone and may have marginal financial resources. Timely referral to a care coordinator or social worker may help to address potential socioeconomic barriers to medication adherence, by allowing him to obtain increased services and better access to the healthcare system.

## Concluding Remarks

The case of Mr. P is illustrative of the challenges concerning medication non-adherence that are faced by clinicians and researchers. As the discussion demonstrates, the use of a robust conceptual model such the one outlined by Fransen et al. (2009) can guide both assessment and treatment of medication non-adherence. Two additional considerations merit discussion. First, it is important to recognize that given the special difficulties of engaging with non-adherent patients, they are very likely to be under-represented in clinical studies that require consent and follow-up, and may be more resistant to interventions that are offered. In addition, non-adherence is multifaceted, and patients who are non-adherent to medications may also be less likely to adhere to other healthy lifestyle choices, creating a negative feedback between non-adherence and deteriorations in motivation, self-efficacy, and the ability to adhere. Thus both the prevalence and adverse impact of medication non-adherence are likely to be underestimated. To fully address this fundamental issue, considerable insights from the field of health psychology will be needed to better engage with these patients, and to discover better methods to detect, understand, and effectively treat medication non-adherence.

## Conflict of Interest Statement

The authors declare that the research was conducted in the absence of any commercial or financial relationships that could be construed as a potential conflict of interest.

## Appendix

**Table A1:** **Outcome of medication adherence assessment for Mr. P**.

Measures	Details	Results
Barriers to adherence	Asks whether prescriptions were filled, and if not, reasons why not (e.g., cost, convenience)	No barriers reported
Morisky scale	8-item self-report medication adherence scale	2/8, indicating poor self-reported medication adherence
Modified CARDIA scale	For each medication, self-reported number of days in the preceding week on which the medication was taken	7/7 for all medications, indicating excellent self-reported medication adherence
Aspirin		
Clopidogrel		
Beta-blocker		
Statin	
Electronic pill bottle	For each medication, the number of days on which pill bottle was opened, divided by total number of days monitored	6/31 (19.4%)
Aspirin		2/31 (6.5%)
Clopidogrel		3/31 (9/7%)
Beta-blocker		0/31 (0%)
Statin		Indicating poor medication adherence
